# Supplementary material for: BRD9 is a druggable component of interferon‐stimulated gene expression and antiviral activity
Source: EMBO Rep. 2021 Aug 16;22(10):e52823. doi: 10.15252/embr.202152823 (PMC8490982; doi:10.15252/embr.202152823)
Supplement: Supplementary file 1 — Expanded View Figures PDF [file EMBR-22-e52823-s002.pdf]

Expanded View Figures

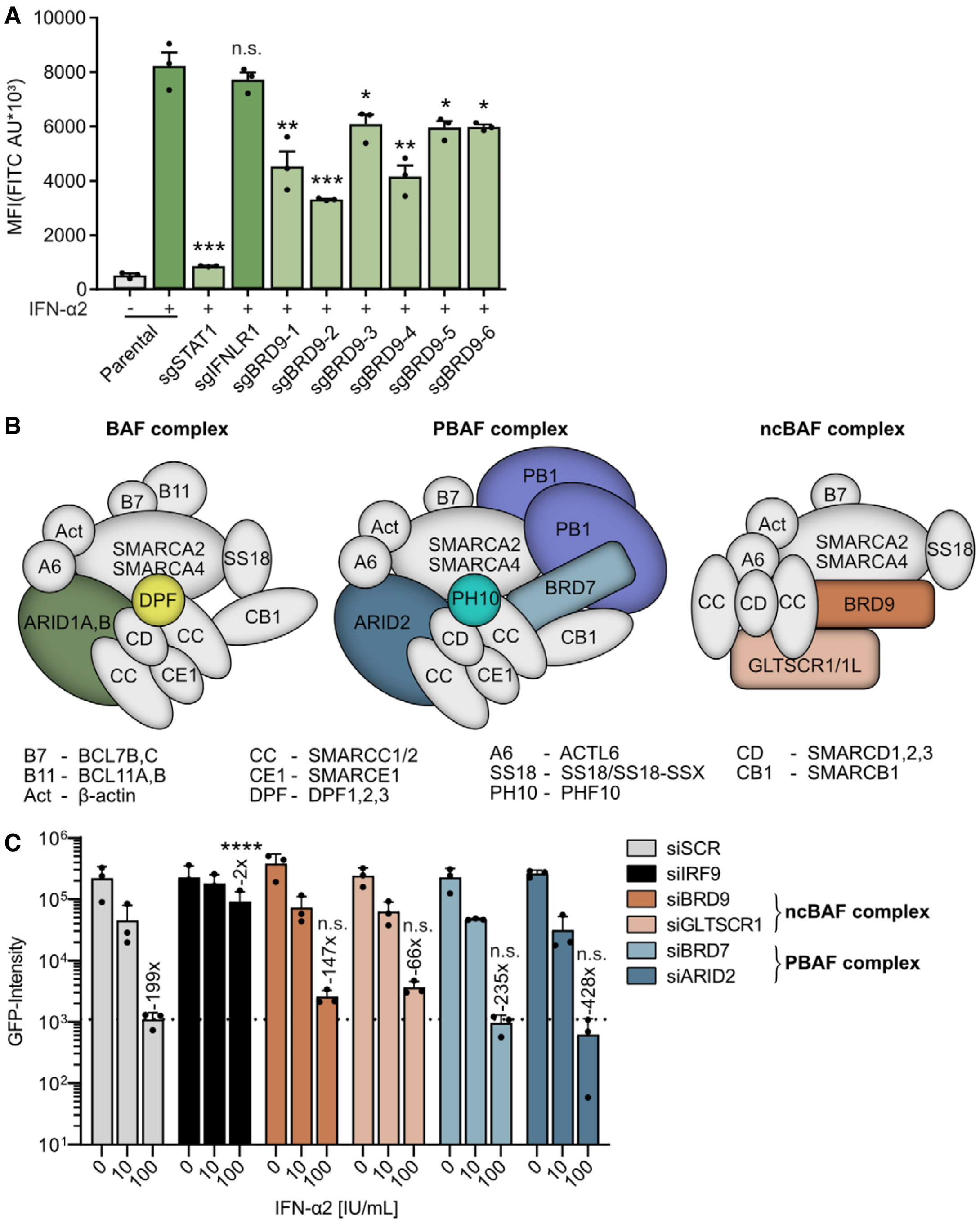

Figure EV1.

© 2021 The Authors EMBO reports 22: e52823 | 2021 19 of 18

◀ **Figure EV1. Independent validation of BRD9 as a hit in the genome-scale screen for factors important for interferon-stimulated gene expression.**

A A549/pr(ISRE).eGFP.A1 cells were transduced for at least 10 days with lentiviruses expressing Cas9 and individual sgRNAs derived from the GeCKOv2 CRISPR-Cas9 library targeting BRD9, STAT1, or IFNLR1. eGFP levels following 16 h of IFN-α2 treatment (1,000 IU/ml), or mock, were determined by flow cytometry. MFI = mean fluorescence intensity. Data represent means and standard deviations from $n = 3$ biological experiments (individual data points shown). Statistical significance was determined relative to the parental cells stimulated with IFN-α2 using single-tailed ANOVA (*$P < 0.05$; **$P < 0.01$; ***$P < 0.001$; n.s. not significant).

B Schematic representations of the known components of the canonical BRG1- or BRM-associated factors (BAF) complex, the Polybromo-containing BAF (PBAF) complex, and the non-canonical BAF (ncBAF) complex. Factors reported to be unique to each complex are indicated with colored shapes. BRD9 is unique to the ncBAF complex.

C A549-2D8 cells were transfected for 32 h with the indicated siRNA SMARTpools, or siSCR control, prior to treatment with 0, 10, or 100 IU/ml of IFN-α2 for 16 h. Cells were then infected with VSV-GFP at an MOI of 1 PFU/cell and total integrated green fluorescent intensities were collected using the Incucyte live-cell analysis system over the course of 24 h. Area under the curve (AUC) values for GFP-Intensity during the 10–24 h period post-infection were determined. Statistical significance was determined by 1-way ANOVA on log-transformed values comparing the siSCR + 100 IU/ml IFN-α2 condition to each of the other + 100 IU/ml IFN-α2 conditions (****$P < 0.0001$; n.s. not significant). Dotted line is a visual guide for minimum virus replication in siSCR cells in the presence of 100 IU/ml IFN-α2. Numbers above IFN-α2-treated bars indicate their approximate difference to the respective untreated conditions. Data represent means and standard deviations from $n = 3$ biological experiments (individual data points shown).

 © 2021 The Authors

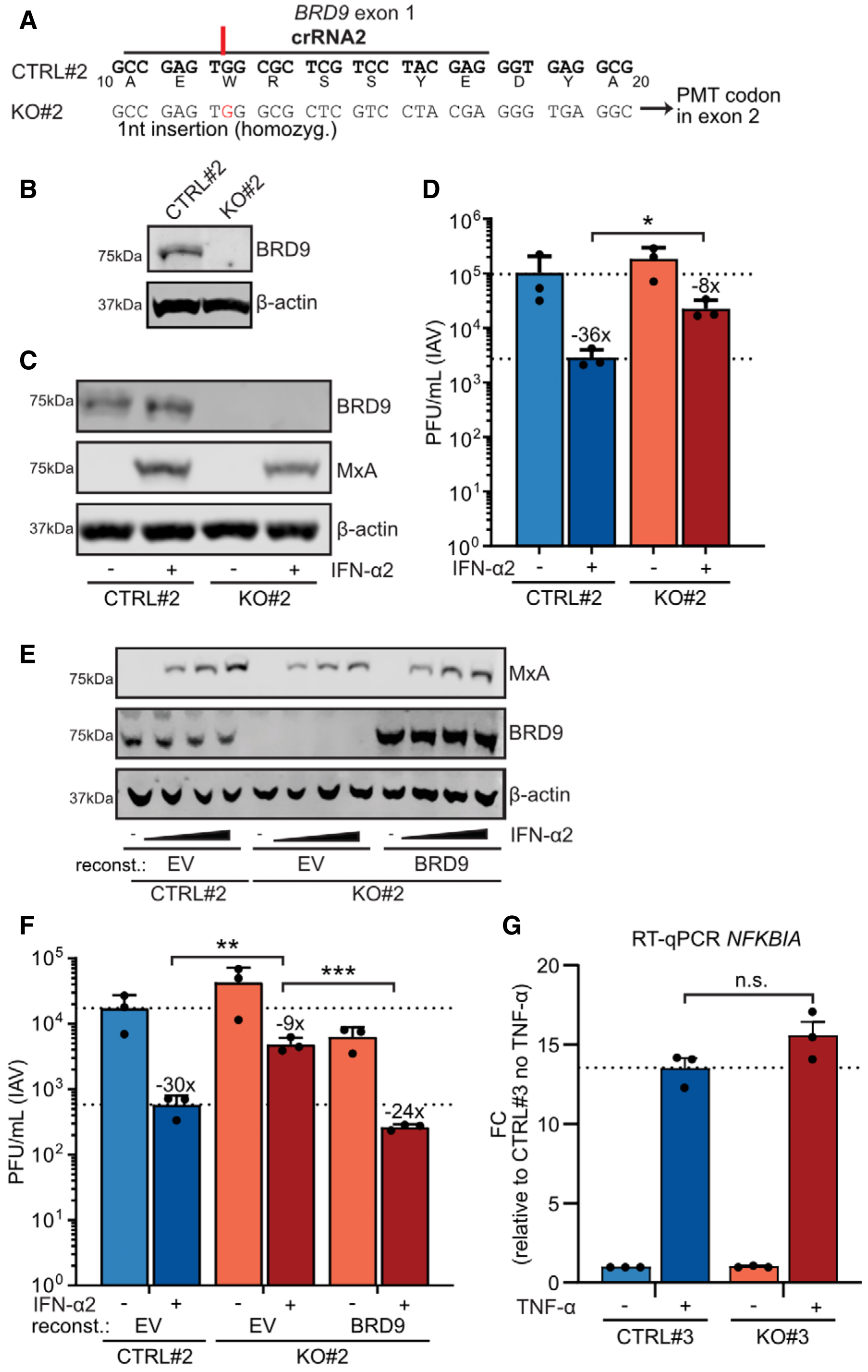

Figure EV2. Data generated using an independent knockout cell clone to validate BRD9 as important for interferon-stimulated antiviral activity.

A An A549-derived BRD9-KO cell clone (KO#2) was generated using a crRNA targeting exon 1 of *BRD9*. The target sequence of the crRNA (termed crRNA2), and the resulting 1nt homozygous genomic insertion determined by NGS, are shown in comparison to an unedited control clone (CTRL#2). The generated insertion leads to a premature termination (PMT) codon in the following exon. Encoded amino-acids are shown below the CTRL nucleotide sequence.

B Western blot analysis of lysates from CTRL#2 or BRD9-KO#2 cells. BRD9 and β-actin were detected with specific antibodies. Data are representative of at least two biological replicates.

C Western blot analysis of CTRL#2 or BRD9-KO#2 lysates from cells treated for 16 h with 1,000 IU/ml of IFN-α2. The indicated proteins were detected with specific antibodies. Data are representative of at least two biological replicates.

D CTRL#2 or BRD9-KO#2 cells were treated, or not, with 1,000 IU/ml of IFN-α2 for 16 h prior to infection with IAV (WSN/33) at an MOI of 0.01 PFU/cell. Viral titers were determined after 24 h by plaque assay.

E CTRL#2 or BRD9-KO#2 cells were stably transduced with BRD9-expressing, or control (EV, empty vector), lentiviruses and treated with a range of IFN-α2 concentrations (0, 10, 100, 1,000 IU/ml) for 16 h prior to lysis and analysis for the indicated proteins by Western blot. Data are representative of at least two biological replicates.

F CTRL#2 or BRD9-KO#2 cells were stably transduced with BRD9-expressing, or control (EV, empty vector), lentiviruses and treated with 1,000 IU/ml of IFN-α2 for 16 h prior to infection with IAV (WSN/33) at an MOI of 0.01 PFU/cell. Viral titers were determined after 24 h by plaque assay.

G RT–qPCR analysis of *NFKBIA* levels in CTRL#3 or BRD9-KO#3 cells following treatment, or not, with 10 ng/ml of TNF-α for 2 h. *GAPDH* transcript levels were used for normalization. Data represent means and standard deviations of fold expression changes (relative to CTRL#3 without TNF-α treatment) from $n = 3$ biological experiments (individual data points shown). Statistical significance was determined by one-way ANOVA on ΔC_t values (n.s. not significant).

Data information: For (D) and (F), data represent means and standard deviations from $n = 3$ biological experiments (individual data points shown). Statistical significance was determined by 1-way ANOVA on log-transformed plaque counts (*$P < 0.05$; **$P < 0.01$; ***$P < 0.001$). Dotted lines are a visual guide for maximum and minimum virus replication in control cells in the absence and presence of IFN-α2, respectively. Numbers above IFN-α2-treated bars indicate their approximate difference to the respective untreated conditions.

Source data are available online for this figure.

© 2021 The Authors

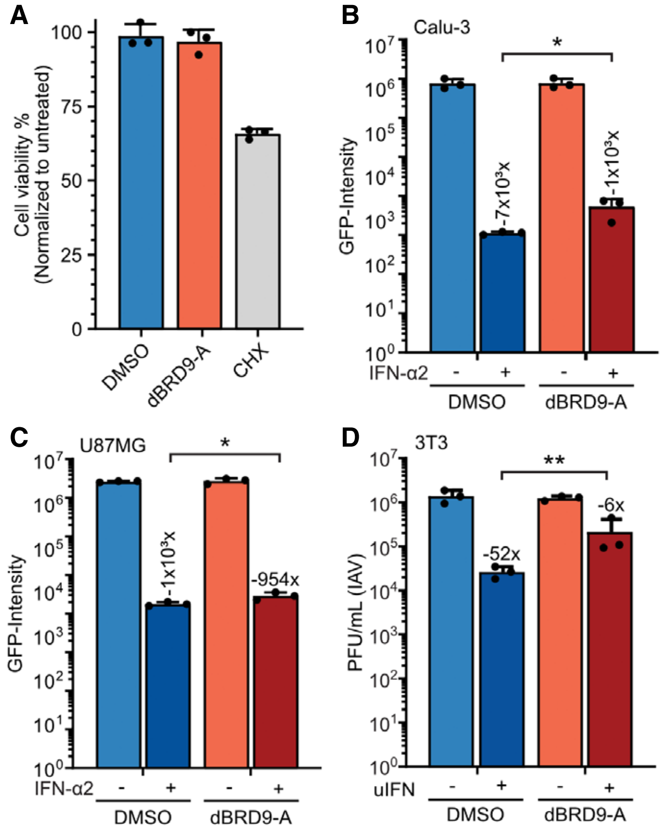

Figure EV3. Targeted degradation of BRD9 is non-cytotoxic and reveals its cell type-independent contribution to interferon-stimulated antiviral activity.

A A549 cells were treated for 22 h with either DMSO, 125 nM dBRD9-A, or 1 μg/ml cycloheximide (CHX). CellTiterGlo was used to determine cell viability relative to untreated cells. Data represent means and standard deviations from $n = 3$ biological experiments (individual data points shown).

B–D Calu-3 (B), U87MG (C), or 3T3 (D) cells were treated with 125 nM dBRD9-A or DMSO for 6 h prior stimulation with 1,000 IU/ml of IFN-α2 (B-C) or 400 IU/ml of universal type I IFN (D) for 16 h. Cells were then infected with VSV-GFP at an MOI of 0.6 PFU/cell and total integrated green fluorescent intensities were determined using the Incucyte live-cell analysis system at 24 h post-infection (B-C). Data represent means and standard deviations from $n = 3$ biological experiments (individual data points shown). Statistical significance was determined by 1-way ANOVA on log-transformed intensity values ($^*P < 0.05$). For (D), cells were infected with IAV (WSN/33) at an MOI of 0.001 PFU/cell. Viral titers were determined after 52 h by plaque assay. Data represent means and standard deviations from $n = 3$ biological experiments (individual data points shown). Statistical significance was determined by 1-way ANOVA on log-transformed plaque counts ($^{**}P$-value < 0.01). Numbers above IFN-treated bars indicate their approximate difference to the respective untreated conditions.

© 2021 The Authors

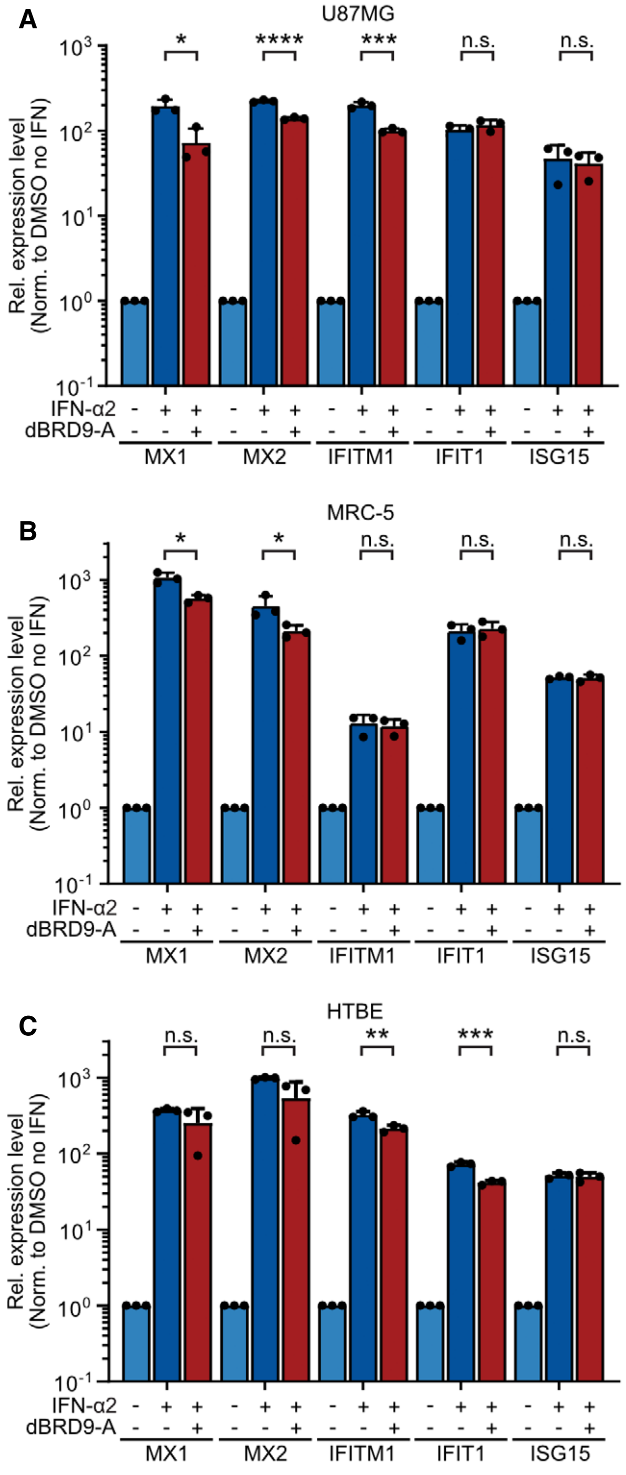

Figure EV4. Cell-type specific interferon-stimulated gene subsets are affected by dBRD9-A pretreatment.

A–C RT–qPCR results of selected transcripts (*MX1*, *MX2*, *IFITM1*, *IFIT1*, and *ISG15*) in U87MG cells (A), primary-like MRC-5 cells (B), and undifferentiated HTBE cells (C). Cells were pretreated with 125 nM dBRD9-A or DMSO for 6 h prior to induction of ISG transcription by addition of 100 IU/ml IFN-α2 for 6 h. *GAPDH* transcript levels were used for normalization. Data represent means and standard deviations from $n = 3$ biological experiments (individual data points shown). Statistical significance was determined by unpaired 2-tailed *t*-test on ΔC_t values (**P*-value < 0.05, ***P*-value < 0.01, ****P*-value < 0.001, *****P*-value < 0.0001, n.s. not significant).

© 2021 The Authors

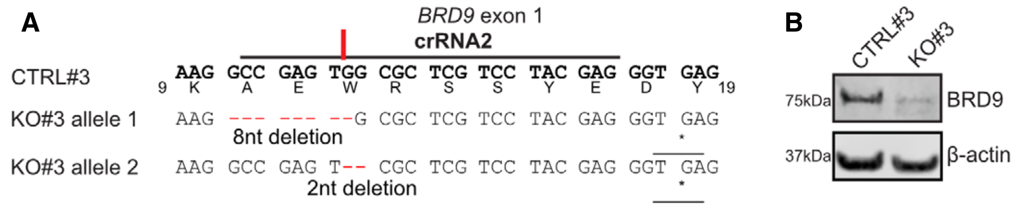

Figure EV5. An independent BRD9 knockout cell clone derived from a highly IFN-responsive A549 sub-clone.

A An A549-2D8-derived BRD9-KO cell clone (KO#3) was generated using a crRNA targeting exon 1 of *BRD9*. The target sequence of the crRNA (termed crRNA2), and the resulting 8nt and 2nt heterozygous genomic deletions determined by NGS, is shown in comparison to an unedited control clone (CTRL#3), also derived from A549-2D8. The generated deletions lead to premature termination codons indicated with an asterisk (*). Encoded amino acids are shown below the CTRL nucleotide sequence.

B Western blot analysis of lysates from CTRL#3 or BRD9-KO#3 cells. BRD9 and β-actin were detected with specific antibodies. Data are representative of at least two biological replicates.

Source data are available online for this figure.

 © 2021 The Authors
