# Supplementary figures and images for: BRD9 is a druggable component of interferon‐stimulated gene expression and antiviral activity
Source: EMBO Rep. 2021 Aug 16;22(10):e52823. doi: 10.15252/embr.202152823 (PMC8490982; doi:10.15252/embr.202152823)

Figure EV2 - western blot source data

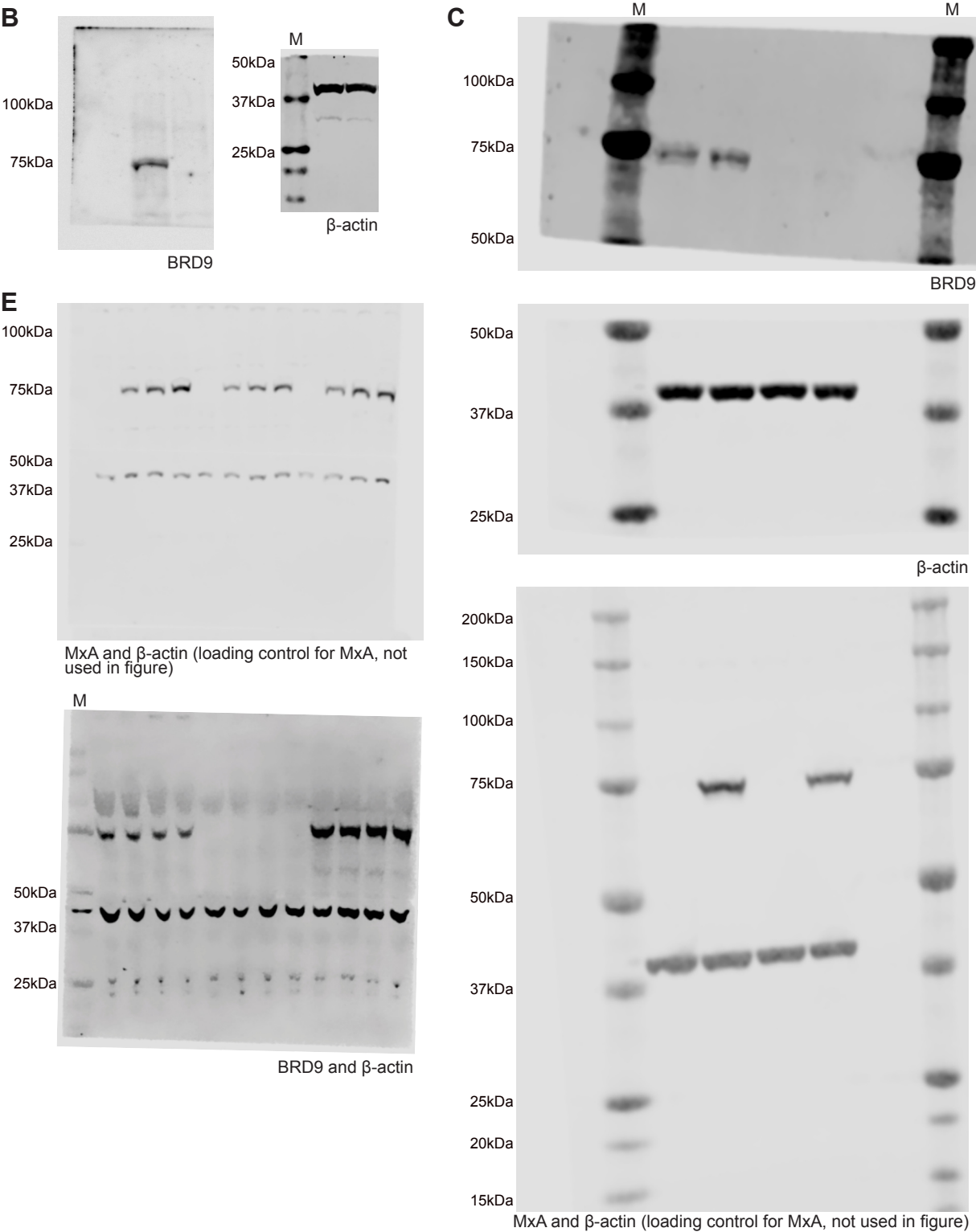

Supplement: Supplementary file 6 — Source Data for Expanded View [file EMBR-22-e52823-s010.zip › 52823-ev-source-data/EMBOR-2021-52823V3-Figure_EV2_Source_Data-sd.pdf]

Figure EV5 - western blot source data

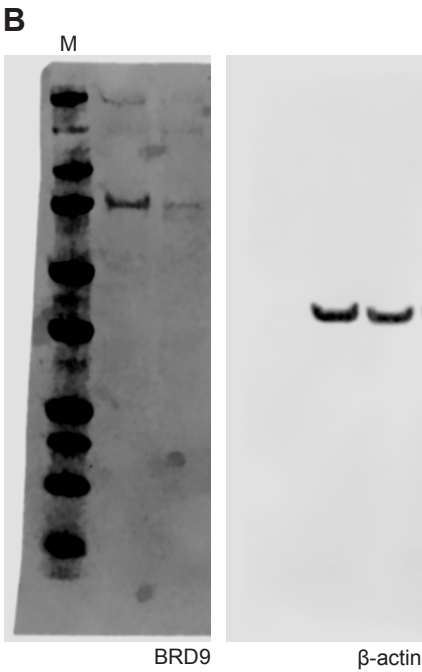

Supplement: Supplementary file 6 — Source Data for Expanded View [file EMBR-22-e52823-s010.zip › 52823-ev-source-data/EMBOR-2021-52823V3-Figure_EV5_Source_Data-sd.pdf]

Figure 2 - western blot source data

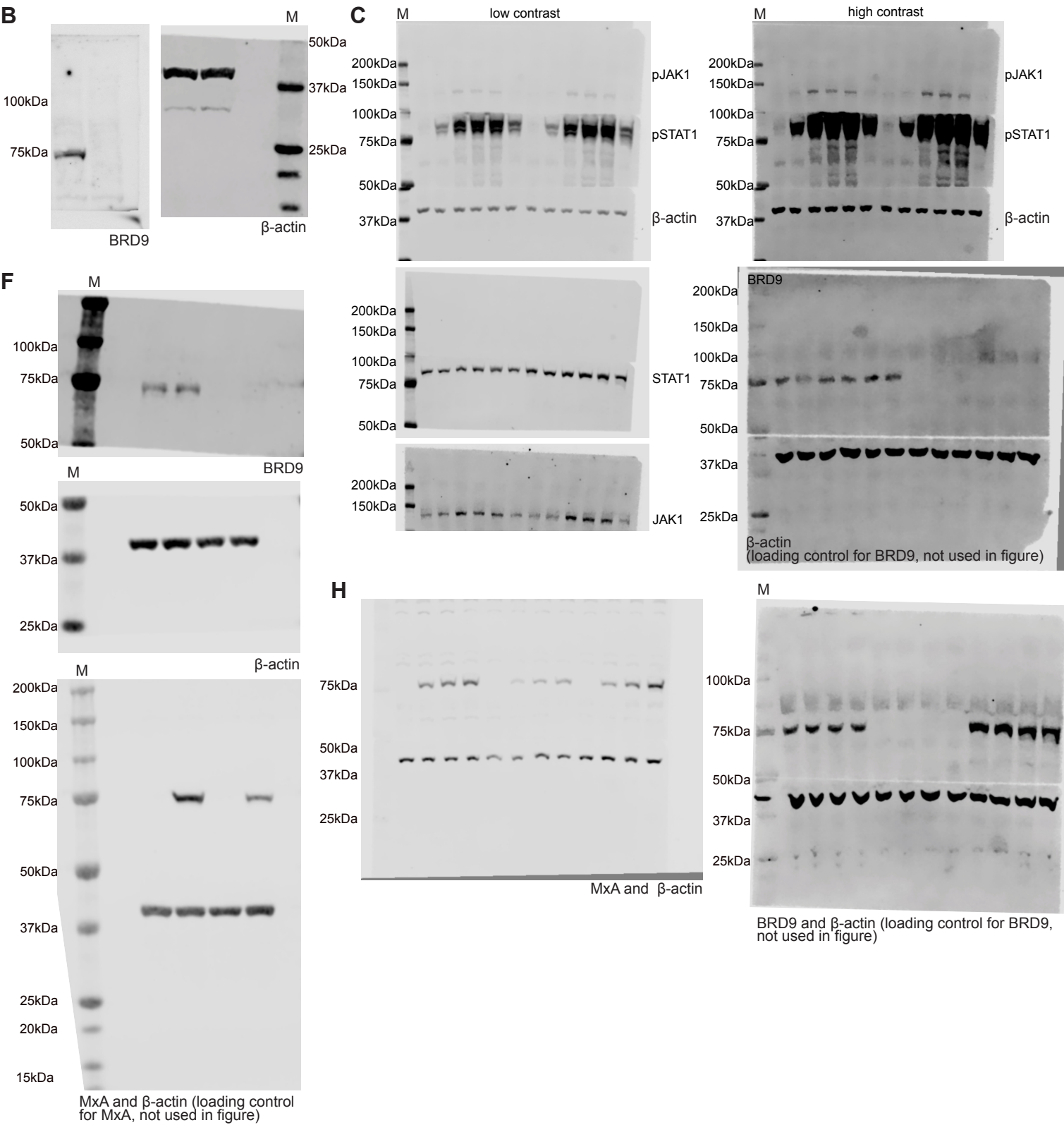

Supplement: Supplementary file 7 — Source Data for Figure 2 [file EMBR-22-e52823-s007.pdf]

Figure 3 - western blot source data

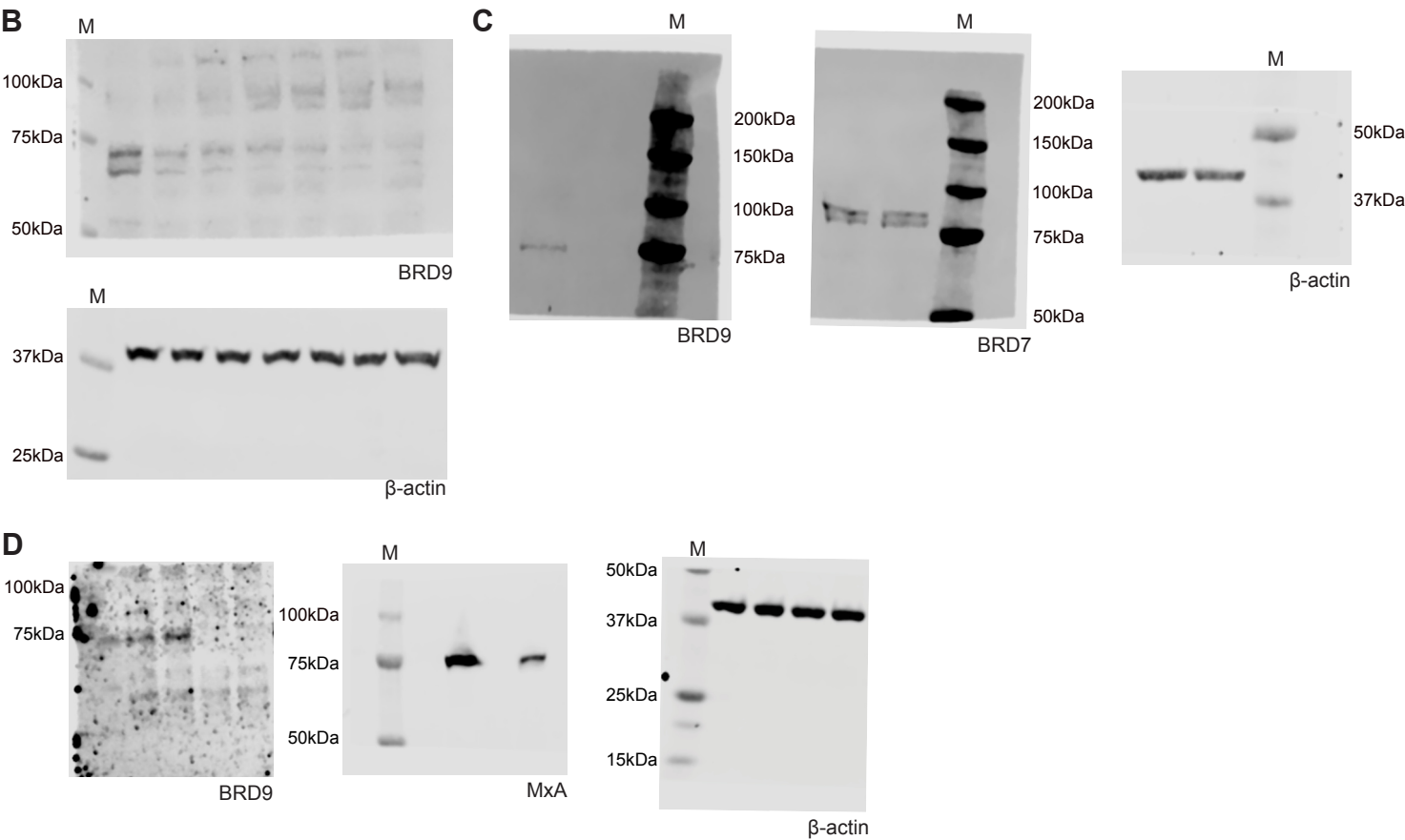

Supplement: Supplementary file 8 — Source Data for Figure 3 [file EMBR-22-e52823-s003.pdf]

Figure 5 - western blot source data

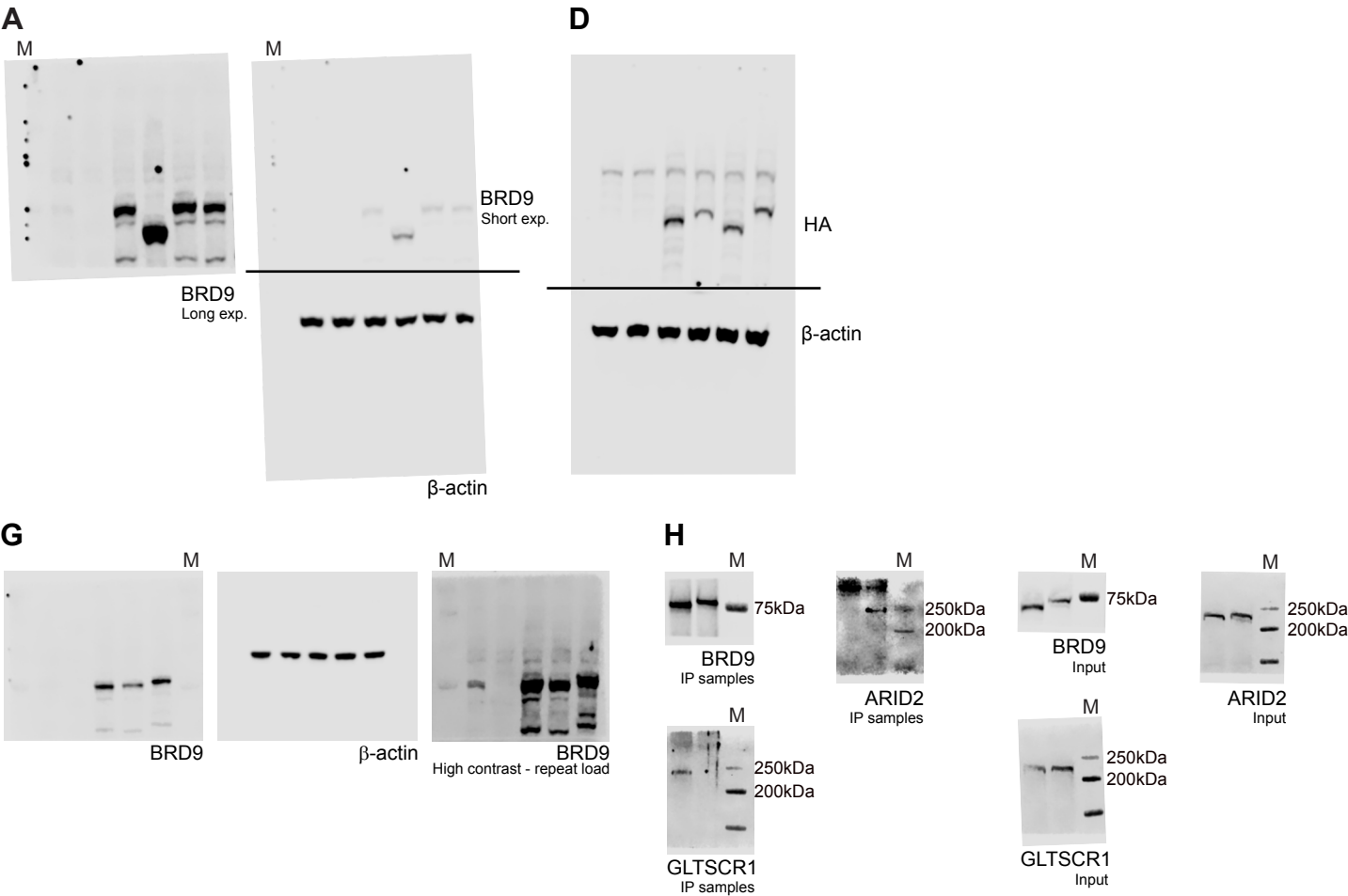

Supplement: Supplementary file 9 — Source Data for Figure 5 [file EMBR-22-e52823-s009.pdf]

Figure 6 - western blot source data

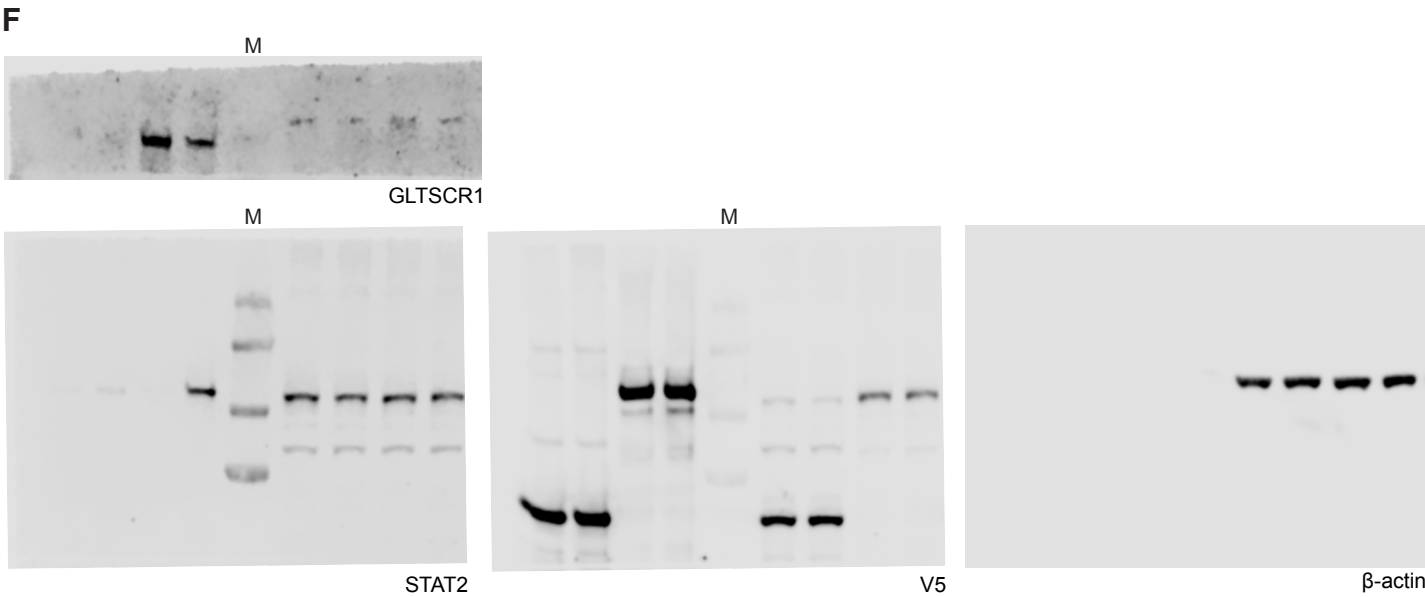

Supplement: Supplementary file 10 — Source Data for Figure 6 [file EMBR-22-e52823-s001.pdf]
