# Supplementary material for: BRD9 is a druggable component of interferon‐stimulated gene expression and antiviral activity
Source: EMBO Rep. 2021 Aug 16;22(10):e52823. doi: 10.15252/embr.202152823 (PMC8490982; doi:10.15252/embr.202152823)
Supplement: Supplementary file 11 [file EMBR-22-e52823-s011.pdf]

EMBO reports

BRD9 is a Druggable Component of Interferon-Stimulated Gene Expression and Antiviral Activity

Jacob Börold, Davide Eletto, Idoia Busnadiego, Nina Mair, Eva Moritz, Samira Schiefer, Nora Schmidt, Philipp Petric, W. Wong, Martin Schwemmle, and Benjamin Hale
DOI: 10.15252/embr.202152823

Corresponding author(s): Benjamin Hale (hale.ben@virology.uzh.ch)

Review Timeline:

Submission Date:	9th Mar 21
Editorial Decision:	8th Apr 21
Revision Received:	1st Jul 21
Editorial Decision:	20th Jul 21
Revision Received:	23rd Jul 21
Accepted:	27th Jul 21

Editor: Achim Breiling

Transaction Report:

(Note: With the exception of the correction of typographical or spelling errors that could be a source of ambiguity, letters and reports are not edited. Depending on transfer agreements, referee reports obtained elsewhere may or may not be included in this compilation. Referee reports are anonymous unless the Referee chooses to sign their reports.)

1st Editorial Decision 8th Apr 2021

Dear Prof. Hale,

Thank you for the submission of your research manuscript to EMBO reports. We have now received the reports from the three referees that were asked to evaluate your study, which can be found at the end of this email.

As you will see, the referees think that these findings are of interest. However, they have several comments, concerns and suggestions, indicating that a major revision of the manuscript is necessary to allow publication of the study in EMBO reports. As the reports are below, and all their points need to be addressed, I will not detail them here.

Given the constructive referee comments, we would like to invite you to revise your manuscript with the understanding that all referee concerns must be addressed in the revised manuscript or in the detailed point-by-point response. Acceptance of your manuscript will depend on a positive outcome of a second round of review. It is EMBO reports policy to allow a single round of revision only and acceptance of the manuscript will therefore depend on the completeness of your responses included in the next, final version of the manuscript.

Revised manuscripts should be submitted within three months of a request for revision. We are aware that many laboratories cannot function at full efficiency during the current COVID-19/SARS-CoV-2 pandemic and we have therefore extended our 'scooping protection policy' to cover the period required for full revision. Please contact me to discuss the revision should you need additional time, and also if you see a paper with related content published elsewhere.

When submitting your revised manuscript, please also carefully review the instructions that follow below.

PLEASE NOTE THAT upon resubmission revised manuscripts are subjected to an initial quality control prior to exposition to re-review. Upon failure in the initial quality control, the manuscripts are sent back to the authors, which may lead to delays. Frequent reasons for such a failure are the lack of the data availability section (please see below) and the presence of statistics based on n=2 (the authors are then asked to present scatter plots or provide more data points).

When submitting your revised manuscript, we will require:

1) a .docx formatted version of the final manuscript text (including legends for main figures, EV figures and tables), but without the figures included. Please make sure that changes are highlighted to be clearly visible. Figure legends should be compiled at the end of the manuscript text.

2) individual production quality figure files as .eps, .tif, .jpg (one file per figure), of main figures and EV figures. Please upload these as separate, individual files upon re-submission.

The Expanded View format, which will be displayed in the main HTML of the paper in a collapsible format, has replaced the Supplementary information. You can submit up to 5 images as Expanded View. Please follow the nomenclature Figure EV1, Figure EV2 etc. The figure legend for these should be included in the main manuscript document file in a section called Expanded View Figure Legends after the main Figure Legends section. Additional Supplementary material should be supplied as a single pdf file labeled Appendix. The Appendix should have page numbers and needs

to include a table of content on the first page (with page numbers) and legends for all content. Please follow the nomenclature Appendix Figure Sx, Appendix Table Sx etc. throughout the text, and also label the figures and tables according to this nomenclature.

For more details, please refer to our guide to authors:
http://www.embopress.org/page/journal/14693178/authorguide#manuscriptpreparation

See also our guide for figure preparation:
http://wol-prod-cdn.literatumonline.com/pb-assets/embo-site/EMBOPress_Figure_Guidelines_061115-1561436025777.pdf

3) a .docx formatted letter INCLUDING the reviewers' reports and your detailed point-by-point responses to their comments. As part of the EMBO Press transparent editorial process, the point-by-point response is part of the Review Process File (RPF), which will be published alongside your paper.

4) a complete author checklist, which you can download from our author guidelines (https://www.embopress.org/page/journal/14693178/authorguide). Please insert page numbers in the checklist to indicate where the requested information can be found in the manuscript. The completed author checklist will also be part of the RPF.

Please also follow our guidelines for the use of living organisms, and the respective reporting guidelines: http://www.embopress.org/page/journal/14693178/authorguide#livingorganisms

5) that primary datasets produced in this study (e.g. RNA-seq, ChIP-seq, structural and array data) are deposited in an appropriate public database. If no primary datasets have been deposited, please also state this a dedicated section (e.g. 'No primary datasets have been generated and deposited'), see below.

See also: http://embor.embopress.org/authorguide#datadeposition

Please remember to provide a reviewer password if the datasets are not yet public.

The accession numbers and database should be listed in a formal "Data Availability " section (placed after Materials & Methods) that follows the model below. This is now mandatory (like the COI statement). Please note that the Data Availability Section is restricted to new primary data that are part of this study.

Data availability

The datasets produced in this study are available in the following databases:

- RNA-Seq data: Gene Expression Omnibus GSE46843 (https://www.ncbi.nlm.nih.gov/geo/query/acc.cgi?acc=GSE46843)
- [data type]: [name of the resource] [accession number/identifier/doi] ([URL or identifiers.org/DATABASE:ACCESSION])

*** Note - All links should resolve to a page where the data can be accessed. ***

Moreover, I have these editorial requests:

6) We strongly encourage the publication of original source data with the aim of making primary data more accessible and transparent to the reader. The source data will be published in a separate source data file online along with the accepted manuscript and will be linked to the relevant figure. If you would like to use this opportunity, please submit the source data (for example scans of entire gels or blots, data points of graphs in an excel sheet, additional images, etc.) of your key experiments together with the revised manuscript. If you want to provide source data, please include size markers for scans of entire gels, label the scans with figure and panel number, and send one PDF file per figure.

7) Our journal encourages inclusion of *data citations in the reference list* to directly cite datasets that were re-used and obtained from public databases. Data citations in the article text are distinct from normal bibliographical citations and should directly link to the database records from which the data can be accessed. In the main text, data citations are formatted as follows: "Data ref: Smith et al, 2001" or "Data ref: NCBI Sequence Read Archive PRJNA342805, 2017". In the Reference list, data citations must be labeled with "[DATASET]". A data reference must provide the database name, accession number/identifiers and a resolvable link to the landing page from which the data can be accessed at the end of the reference. Further instructions are available at:
http://www.embopress.org/page/journal/14693178/authorguide#referencesformat

8) Regarding data quantification and statistics, can you please specify, where applicable, the number "n" for how many independent experiments (biological replicates) were performed, the bars and error bars (e.g. SEM, SD) and the test used to calculate p-values in the respective figure legends. Please provide statistical testing where applicable, and also add a paragraph detailing this to the methods section. See:
http://www.embopress.org/page/journal/14693178/authorguide#statisticalanalysis

9) Please also note our new reference format:
http://www.embopress.org/page/journal/14693178/authorguide#referencesformat

10) For microscopic images, please add scale bars of similar style and thickness to all the microscopic images, using clearly visible black or white bars (depending on the background). Please place these in the lower right corner of the images. Please do not write on or near the bars in the image but define the size in the respective figure legend.

11) Please also add up to 5 keywords to the title page.

I look forward to seeing a revised version of your manuscript when it is ready. Please let me know if you have questions or comments regarding the revision.

Yours sincerely,

Achim Breiling
Editor
EMBO Reports

Referee #1:

In the manuscript by Börold et al., entitled "Genome-Wide CRISPR Screening Identifies BRD9 as a

Druggable Component of Interferon-Stimulated Gene Expression and Antiviral Activity", authors identify and characterize a previously unappreciated component of IFN-a signaling chain, bromodomain-containing protein 9 (BRD9), which they identified using a genome-wide CRISPR/Cas9 loss-of-function screen. In in vitro experiments, using a BRD9 knock-out cell line and pharmacological inhibition of BRD9 authors convincingly demonstrate that this protein is involved in transcriptional activation of a subset of ISGs (which somewhat differed among the different cell lines tested) upon IFN-a2 stimulation and is crucial for maximizing the effect of IFN-a2 against a range of viruses and in a variety of cell lines. Authors then go to great lengths to functionally characterize BRD9 in order to get a mechanistic insight on its function. Finally, authors hypothesize that since BRD9 can be specifically targeted by small molecules and it only affects a subset of ISGs it might be considered as a potentially safer target in treatment of certain immunopathological conditions. Therefore, current study warrants further in vivo work to demonstrate the relevance of BRD9 for IFN-a signaling in a more physiological context (not as a part of the revision).

The study is of very high quality, experiments are well done and the results support the conclusions of the authors. The main finding is novel and provides a new angle on the regulation of transcriptional response to type-I IFNs, and therefore would be of interest to everybody working in the innate immunity and cytokine signaling field. The only "weak" side is that all the work has been done in vitro/cell culture and therefore the physiological relevance of the findings remains unclear at the moment

The discussion is focused mainly on the mechanistic aspects of BRD9 activity and could include some of the more general aspects for example the relevance of such a mechanism of additional transcriptional regulation in the context of IFN (and perhaps other cytokine) signaling (most ISGs don't need it, and on its own BRD9 inhibition does not seem to have a strong effect on the cells in the absence of IFN-a, at least short-term), gene-specificity of BRD9 and why BRD9 appears to be not important in type-III IFN signaling etc.

I only have some minor points/suggestions:

1. Fig. 2E - the fold-change values are not informative here, because a) the baseline expression of Mx1 is not known in different cell lines and b) the maximum levels of Mx1 induction would be more relevant and easy to correlate with subsequent antiviral effect, therefore actual gene expression values should be shown instead.

2. Fig. 2G,I, Supp Fig. 2F, Fig. 3E-L, Supp Fig. 3B-D - contrary to my previous comment, here fold change/% inhibition (using virus titer/GFP intensity in untreated cells as 100%) values would be more appropriate and easier to compare between BRD9+/- or dBRD9-A+/- conditions

3. Fig. 2G,I (and Supp), Fig. 3E-L (and Supp) - what are the dotted lines?

4. Why did the authors choose 16h time point for their IFN-a2 stimulation experiments? IFN activity could be already waning after 16h due to early induction of negative regulators of JAK/STAT signaling (USP18, SOCS1/3)

5. Are there any naturally occurring polymorphisms of BRD9 and if yes how do they affect immune response in the carriers (could be a part of discussion)

Referee #2:

In the manuscript entitled "Genome-Wide CRISPR 1 Screening Identifies BRD9 as a Druggable Component of Interferon-Stimulated Gene Expression and Antiviral Activity", Borold et al identified epigenetic reader BRD9 as a novel regulator of the type I interferon response through a genome-wide CRISPR screen, and the authors determine that BRD9 promotes the antiviral state in human cell lines of epithelial, fibroblast, and hepatocyte origin. A previous study from a separate group also identified BRD9 as a positive regulator of the type III interferon response but did not further characterize the role of BRD9. The present study utilized BRD9 knockout cell lines and reconstitutions to prove their hypothesis that BRD9 does not affect the type I IFN signaling pathway yet affects transcription of ISGs. Furthermore, they demonstrate that targeting BRD9 for degradation via a small molecule recapitulate their findings that BRD9 enhances transcription of a subset of interferon response genes. Previous research has shown that BRD9 associates with the noncanonical BAF chromatin remodeling complex through a DUF3512 scaffolding domain. Therefore, the authors utilize a domain-swapping technique to determine that the BRD9 DUF3512 and thus association with the noncanonical BAF complex is necessary for the BRD9-mediated function in ISG transcription. This work expands our understanding of the role of the noncanonical BAF complex in the interferon response. Knowledge gained form this paper sets up future work to study the role of BRD9 inhibitors in vivo in inhibiting the interferon response to treat interferonopathies. The following are specific areas for improvement prior to publication:

Major Points:

1. Quantification of Figure 1B and C would help give an idea how consistent this assay is for use in subsequent figures.

2. Is the phenotype observed in Fig 2 and 3 only present when IFN-a2 added?

3. What is determining the specificity of BRD9 to those specific ISG subsets since it is mentioned there were no consensus sequences in their promoters.

4. The authors performed RNAseq on IFN-a2 stimulated cells in the presence or absence of a BRD9 degrador to determine the extent to which BRD9 promoted ISG induction (Figure 4). Based on their hypothesis that BRD9 regulates ISGs, the authors only provide an analysis of how the presence or absence of BRD9 affects ISG transcription. However, the authors could analyze their complete set of RNAseq data to determine whether there are non-ISG sets of genes that are affected by a loss of BRD9. The resulting data could provide evidence that BRD9 primarily affects ISG transcription or shed light on other functions of BRD9 at baseline or after IFN-a2 stimulation that may have implications to the clinical relevance of targeting BRD9.

5. It is suggested that a chromatin accessibility mechanism may be at play since signaling changes weren't significantly seen - ATAC to assess chromatin accessibility in the KOs would specifically address this. Alternatively, revising lines 350-356 in the Discussion to reflect a more reserved interpretation of the findings and suggest BRD9 recruitment to ISGs may affect chromatin accessibility or STAT2 binding or activity at these sites.

6. In regard to Figure 6, the authors could further validate the BRD9 interactome via traditional co-immunoprecipitations if antibodies are available. They could include the HA-BRD9 versus HA-BRD7c mutant as well to confirm the important role of the BRD9 DUF3512 domain in mediating noncanonical BAF complex interactions that is necessary for STAT2 binding. Alternatively, the

authors could perform IPs in the presence or absence of BRD9 degrador.

Minor Points:
(i) In all the figure labels for BRD9 KO experiments, there is ctrl#1 and KO#1 written? Were multiple clones generated and how consistent was this phenotype in other clones?
(ii) In Fig 6F, its shown that BRD9 only binds to STAT2 with IFN-a2. Is this preceded/or concurrent with BRD9 binding with Smarca2?
(iii) In Figure 5B, the authors should change the statistical analysis to compare the mutant BRD9 constructs to the empty vector as they showed in Figure 5D, F.
(iv) How much are the binding proteins of BRD9 affecting its function? I.e. GLTSCR1/1L or Smarca2? If you were to knockdown these genes in KOs or chemically degraded BRD9, do these change the results in Figure 2 and 3?

Referee #3:

The manuscript by Börold et. al. claims that BRD9, a subunit of a chromatin remodeler complex ncBAF is a cofactor of ISG expression and has antiviral activity. The authors pulled out BRD9 from genome-wide loss-of-function crispr screening to identify factors important for IFN signaling. Utilizing both selective knock down and small molecule mediated degradation of BRD9, they confirm the involvement of BRD9 in IFN signaling in multiple cell-types, and antiviral activities against both RNA and DNA viruses. The authors went on to imply that BRD9 acts at the transcription of subsets of ISGs by being recruited to the promoter by its association with STAT2 and exerting its chromatin remodeling activity to mediate full activation.

The strength of the paper is that the execution of the screen and hit validation were thorough and well controlled. Although the loss of function effect of BRD9 on IFN signaling and anti-viral activities are rather weak, rigorous testing through multiple cell types and multiple viruses convinces its role.

Conceptually, however, the finding lacks a novelty and significance. The chromatin remodeling role of BRD9 as a subunit of SWI/SNF complex and its direct role in regulating transcription is rather well established. Thus, the loss of BRD9 limiting ISG expression and in turn reducing antiviral activity are easily expected.

Both proteomic interactome analysis and the chimeric protein reconstitution experiments fell short of providing mechanistical explanation to how BRD9 enhances selective sets of ISG transcription upon IFN response.

However, if the authors can demonstrate that both the interaction to STAT2 and acetyl-binding bromodomain function of BRD9 are responsible of ncBAF (BRD9) recruitment to the promoter of the subsets of ISGs that are selectively effected upon the loss of BRD9, this will elevate the significance of manuscript greatly and would merit further consideration.

Author response letter for EMBOR-2021-52823-T

We thank all the referees for their constructive comments and overall positive assessment of our manuscript. Please find below all the points raised, with our responses indicated in blue italics.

Referee #1:

In the manuscript by Börold et al., entitled "Genome-Wide CRISPR Screening Identifies BRD9 as a Druggable Component of Interferon-Stimulated Gene Expression and Antiviral Activity", authors identify and characterize a previously unappreciated component of IFN-a signaling chain, bromodomain-containing protein 9 (BRD9), which they identified using a genome-wide CRISPR/Cas9 loss-of-function screen. In in vitro experiments, using a BRD9 knock-out cell line and pharmacological inhibition of BRD9 authors convincingly demonstrate that this protein is involved in transcriptional activation of a subset of ISGs (which somewhat differed among the different cell lines tested) upon IFN-a2 stimulation and is crucial for maximizing the effect of IFN-a2 against a range of viruses and in a variety of cell lines. Authors then go to great lengths to functionally characterize BRD9 in order to get a mechanistic insight on its function. Finally, authors hypothesize that since BRD9 can be specifically targeted by small molecules and it only affects a subset of ISGs it might be considered as a potentially safer target in treatment of certain immunopathological conditions. Therefore, current study warrants further in vivo work to demonstrate the relevance of BRD9 for IFN-a signaling in a more physiological context (not as a part of the revision).

The study is of very high quality, experiments are well done and the results support the conclusions of the authors. The main finding is novel and provides a new angle on the regulation of transcriptional response to type-I IFNs, and therefore would be of interest to everybody working in the innate immunity and cytokine signaling field. The only "weak" side is that all the work has been done in vitro/cell culture and therefore the physiological relevance of the findings remains unclear at the moment

> We thank the referee for their very positive assessment of the quality of our study and its novelty to the innate immunity and cytokine signaling fields. We fully agree that future work, beyond the scope of the present study, should address these findings in vivo.

The discussion is focused mainly on the mechanistic aspects of BRD9 activity and could include some of the more general aspects for example the relevance of such a mechanism of additional transcriptional regulation in the context of IFN (and perhaps other cytokine) signaling (most ISGs don't need it, and on its own BRD9 inhibition does not seem to have a strong effect on the cells in the absence of IFN-a, at least short-term), gene-specificity of BRD9 and why BRD9 appears to be not important in type-III IFN signaling etc.

> We thank the referee for their suggestions to improve our Discussion, and have now updated the relevant sections to briefly highlight some of these important questions which should be addressed in future studies (page 18, lines 392-396). In particular, we have now highlighted some screening results from Lumb et al, Cell Reports, 2017, which were not followed up by these investigators, but which suggested a role for GLTSCR1 and BRD9 in type III IFN signaling, demonstrating that the role of ncBAF probably extends to type III IFN signaling (page 18, lines 398-400).

I only have some minor points/suggestions:

1. Fig. 2E - the fold-change values are not informative here, because a) the baseline expression of Mx1 is not known in different cell lines and b) the maximum levels of Mx1 induction would be more relevant and easy to correlate with subsequent antiviral effect, therefore actual gene expression values should be shown instead.

> We thank the referee for highlighting this point. We believe that we have presented the data in the most informative way (all RT-qPCR data in this panel are made relative to the CTRL cell-line in the absence of IFN). However, we realize that we had not labeled this clearly on the original figure panel, which may have led the reader to assume that the data represented fold-change in each cell-line +/- IFN (which would indeed not account for possible baseline differences between the cell-lines as the referee correctly states). We have now updated the y-axis label to clarify this.

2. Fig. 2G,I, Supp Fig. 2F, Fig. 3E-L, Supp Fig. 3B-D - contrary to my previous comment, here fold change/% inhibition (using virus titer/GFP intensity in untreated cells as 100%) values would be more appropriate and easier to compare between BRD9+/- or dBRD9-A+/- conditions

> For complete clarity, we have now also added fold-change inhibition values to the graphs in question so that the reader has all the relevant information. We have also done this for the other figures in the manuscript.

3. Fig. 2G,I (and Supp), Fig. 3E-L (and Supp) - what are the dotted lines?

> These dotted lines are simply to aid the reader visually in seeing maximum and minimum virus replication in control cells +/- IFN. We have now added a statement to this effect in the appropriate figure legends.

4. Why did the authors choose 16h time point for their IFN-a2 stimulation experiments? IFN activity could be already waning after 16h due to early induction of negative regulators of JAK/STAT signaling (USP18, SOCS1/3)

> We have found that this timepoint leads to the largest effect of IFN antiviral activity (as compared to earlier timepoints), and is therefore very good for the viral assays in this study. For RT-qPCR (gene expression) assays we have used earlier timepoints as indicated in the figure legends.

5. Are there any naturally occurring polymorphisms of BRD9 and if yes how do they affect immune response in the carriers (could be a part of discussion)

> This is a very interesting point and currently something that we are addressing experimentally with the aim of putting together a follow-up manuscript shortly. We therefore prefer not to add this to the discussion presently.

Referee #2:

In the manuscript entitled "Genome-Wide CRISPR 1 Screening Identifies BRD9 as a Druggable Component of Interferon-Stimulated Gene Expression and Antiviral Activity", Borold et al identified epigenetic reader BRD9 as a novel regulator of the type I interferon response through a genome-wide CRISPR screen, and the authors determine that BRD9 promotes the antiviral state in human cell lines of epithelial, fibroblast, and hepatocyte origin. A previous study from a separate group also identified BRD9 as a positive regulator of the type III interferon response but did not further characterize the role of BRD9. The present study utilized BRD9 knockout cell lines and reconstitutions to prove their hypothesis that BRD9 does not affect the type I IFN signaling pathway yet affects transcription of ISGs. Furthermore, they demonstrate that targeting BRD9 for degradation via a small molecule recapitulate their findings that BRD9 enhances transcription of a subset of interferon response genes. Previous research has shown that BRD9 associates with the noncanonical BAF chromatin remodeling complex through a DUF3512 scaffolding domain. Therefore, the authors utilize a domain-swapping technique to determine that the BRD9 DUF3512 and thus association with the noncanonical BAF complex is necessary for the BRD9-mediated function in ISG transcription. This work expands our understanding of the role of the

noncanonical BAF complex in the interferon response. Knowledge gained form this paper sets up future work to study the role of BRD9 inhibitors in vivo in inhibiting the interferon response to treat interferonopathies.

> We thank the referee for their very positive assessment of our study, its novelty for the innate immunity field, and its implications for current knowledge and future work.

The following are specific areas for improvement prior to publication:

Major Points:

1. Quantification of Figure 1B and C would help give an idea how consistent this assay is for use in subsequent figures.

> In these figure panels, essentially the same result was obtained in two independent replicates which we have now tried to make clear in the figure legend. In addition, the data presented in Fig EV1A summarize the MFI values (means, standard deviations and individual datapoints) for 3 independent replicates of practically the same control experiments (parental, sgSTAT1, sgIFNLR1) which we believe can give the reader an appreciation of the consistency of the assay set-up.

2. Is the phenotype observed in Fig 2 and 3 only present when IFN-a2 added?

> This is an important question raised by the reviewer, and the answer is essentially, yes. In almost all of our assays we have assessed the antiviral activity of IFN by monitoring the single-cycle replication of a particular virus, and under these conditions we only ever noted an effect of BRD9 in the presence of IFN. However, as shown in Figs 2G/I, loss of BRD9 did slightly increase IAV replication in the absence of IFN pre-treatment. For these experiments we used fully replication-competent IAV, and assessed viral titres at much later timepoints following multi-cycle replication. We therefore attribute the impact of BRD9 under these conditions to the production and action of endogenously-produced IFN during the infection. To help the reader, we have now added a sentence relating to dBRD9-A treatment having no effect on virus replication in the absence of IFN (Fig 3) to page 10, lines 213-214.

3. What is determining the specificity of BRD9 to those specific ISG subsets since it is mentioned there were no consensus sequences in their promoters.

> This will be a very important thing to address in future studies, but is currently beyond the scope of the present work. As we mention in the manuscript (page 12, 247-253 when discussing Fig 4 and EV4), there is also a degree of cell-type specificity to the ISGs regulated by BRD9. This suggests that it is unlikely to be promoter sequences per se, but rather an epigenetic state of the particular cell involved which determines specificity. These mechanisms and their functional necessities/consequences require further dissection.

4. The authors performed RNAseq on IFN-a2 stimulated cells in the presence or absence of a BRD9 degrador to determine the extent to which BRD9 promoted ISG induction (Figure 4). Based on their hypothesis that BRD9 regulates ISGs, the authors only provide an analysis of how the presence or absence of BRD9 affects ISG transcription. However, the authors could analyze their complete set of RNAseq data to determine whether there are non-ISG sets of genes that are affected by a loss of BRD9. The resulting data could provide evidence that BRD9 primarily affects ISG transcription or shed light on other functions of BRD9 at baseline or after IFN-a2 stimulation that may have implications to the clinical relevance of targeting BRD9.

> In our study we did identify genes that are affected by dBRD9-A treatment in the absence of IFN. Using our cut-off criteria, only 11 non-ISG genes were downregulated by dBRD9-A treatment, and none were upregulated (in Dataset EV2). We have now tried to make this finding clearer in the main manuscript text, and commented that none of the genes affected

are known to be implicated in the IFN signaling pathway. We have now also listed the top five altered genes to make it easier for the reader to see these without referring to the supplementary dataset. In addition, from our own preliminary analysis which we now comment on, 3 of these 11 dBRD9-A regulated genes have been reported to be putative mammalian ISGs, which could support the idea that they are regulated by a basal STAT transcription factor complex together with BRD9 (page 11, lines 231-237). We could not assign a clear pattern to the other dBRD9-A regulated genes.

5. It is suggested that a chromatin accessibility mechanism may be at play since signaling changes weren't significantly seen - ATAC to assess chromatin accessibility in the KOs would specifically address this. Alternatively, revising lines 350-356 in the Discussion to reflect a more reserved interpretation of the findings and suggest BRD9 recruitment to ISGs may affect chromatin accessibility or STAT2 binding or activity at these sites.

> We thank the reviewer for this suggestion and have revised the discussion on page 19, lines 416-419 to reflect a more reserved interpretation.

6. In regard to Figure 6, the authors could further validate the BRD9 interactome via traditional co-immunoprecipitations if antibodies are available. They could include the HA-BRD9 versus HA-BRD7c mutant as well to confirm the important role of the BRD9 DUF3512 domain in mediating noncanonical BAF complex interactions that is necessary for STAT2 binding. Alternatively, the authors could perform IPs in the presence or absence of BRD9 degrador.

> We have now used cells expressing the wt BRD9 and BRD9/BRD7DUF constructs to assay the co-precipitation of GLTSCR1 or ARID2, respectively, and thereby validate that the domain-swaps we made faithfully re-capitulate published data, and also validate at least some of the BRD9 interactome in the absence of IFN treatment. These data are presented in new Fig 5H (and page 14, lines 293-296), and strengthen our hypothesis that BRD9, and its specific co-ordination of ncBAF, is important for effective ISG induction. Our attempts to stably co-precipitate STAT2 with BRD9 have not been successful, and that is why we have tried to be clear throughout the manuscript that the TurboID methodology employed here picks up proximal or transient interactors. Thus, it is likely that BRD9 and STAT2 come close to one another, rather than physically and stably interact, probably at ISG promoters.

Minor Points:

(i) In all the figure labels for BRD9 KO experiments, there is ctrl#1 and KO#1 written? Were multiple clones generated and how consistent was this phenotype in other clones?

> We did indeed generate multiple BRD9 KO clones (and additional wild-type control clones) in order to ensure that the phenotypes were consistent. We additionally reconstituted each KO clone with BRD9 to ensure that the phenotypes we observed could be reverted. The data from ctrl#2 and KO#2 are presented in EV2, and are wholly consistent with the data in Fig 2 with KO#1. We have added a line in the results section to highlight for the reader that we made multiple KO clones (page 8, lines 175-176). In addition, we generated a 3rd KO (KO#3) for use in Figure 5 (now detailed more in the text, pages 12/13, lines 266-273, new Fig EV5) and could also show that the phenotype of this clone is consistent with the phenotypes of KO#1/2, and can be reversed by BRD9 expression.

(ii) In Fig 6F, its shown that BRD9 only binds to STAT2 with IFN-a2. Is this preceded/or concurrent with BRD9 binding with Smarca2?

> Our TurboID analyses identified that SMARCA2 interacts with BRD9 even in unstimulated cells (Dataset EV3 and Fig 6C), while STAT2 is not associated with BRD9 without stimulation, therefore it is likely that the BRD9-SMARCA2 interaction precedes the increased proximity of ncBAF with STAT2. We have now added a statement to page 16, lines 345-346 try and highlight this.

(iii) In Figure 5B, the authors should change the statistical analysis to compare the mutant BRD9 constructs to the empty vector as they showed in Figure 5D, F.

> We have now changed this as suggested.

(iv) How much are the binding proteins of BRD9 affecting its function? I.e. GLTSCR1/1L or Smarca2? If you were to knockdown these genes in KOs or chemically degraded BRD9, do these change the results in Figure 2 and 3?

> We thank the reviewer for this suggestion as we had not considered looking directly into the contribution of other ncBAF complex components, such as SMARCA2 or GLTSCR1/1L, to ISG transcription. In this direction, we have now performed new experiments, presented in Fig EV1C, where we used siRNAs to deplete specific components of the ncBAF complex (BRD9 or GLTSCR1) or the distinct PBAF complex (BRD7 or ARID2) before studying the antiviral effect of IFN. Our results show that only depletion of the ncBAF complex members, BRD9 or GLTSCR1, impact the antiviral effect of IFN, and not depletion of PBAF complex members, BRD7 or ARID2. These new data support the notion that it is the ncBAF complex mediating this effect and not the PBAF complex, which we think is a nice demonstration of specificity in the transcription-regulatory machinery, and provides additional novelty to the paper. These new data also support the BRD9/BRD7 domain swapping experiments presented in Fig 5 that show that BRD9 functions with GLTSCR1/1L. We have discussed these new observations on page 8, lines 164-173.

Referee #3:

The manuscript by Börold et. al. claims that BRD9, a subunit of a chromatin remodeler complex ncBAF is a cofactor of ISG expression and has antiviral activity. The authors pulled out BRD9 from genome-wide loss-of-function crispr screening to identify factors important for IFN signaling. Utilizing both selective knock down and small molecule mediated degradation of BRD9, they confirm the involvement of BRD9 in IFN signaling in multiple cell-types, and antiviral activities against both RNA and DNA viruses. The authors went on to imply that BRD9 acts at the transcription of subsets of ISGs by being recruited to the promoter by its association with STAT2 and exerting its chromatin remodeling activity to mediate full activation.

The strength of the paper is that the execution of the screen and hit validation were thorough and well controlled. Although the loss of function effect of BRD9 on IFN signaling and anti-viral activities are rather weak, rigorous testing through multiple cell types and multiple viruses convinces its role.

> We thank the referee for this very positive assessment of the strengths of our study and the rigorousness of our experimentation.

Conceptually, however, the finding lacks a novelty and significance. The chromatin remodeling role of BRD9 as a subunit of SWI/SNF complex and its direct role in regulating transcription is rather well established. Thus, the loss of BRD9 limiting ISG expression and in turn reducing antiviral activity are easily expected.

Both proteomic interactome analysis and the chimeric protein reconstitution experiments fell short of providing mechanistical explanation to how BRD9 enhances selective sets of ISG transcription upon IFN response. However, if the authors can demonstrate that both the interaction to STAT2 and acetyl-binding bromodomain function of BRD9 are responsible of ncBAF (BRD9) recruitment to the promoter of the subsets of ISGs that are selectively effected upon the loss of BRD9, this will elevate the significance of manuscript greatly and would merit further consideration.

> We are disappointed that the 3rd referee did not appreciate the novelty or significance of our study to the innate immunity and cytokine signaling fields. While the reviewer is correct that BRD9 is already known to play a role in transcription, we believe that several aspects of our original submission, as well as new experiments performed for this revision (outlined below), do indeed provide novel and valuable information for both the virology and innate immunity communities. The reviewer is also correct that it would be of great interest in the future to determine the recruitment mechanism of BRD9/ncBAF and STAT2/ISGF3 to specific ISG promoters, and determine if an interaction between the two (perhaps acetyl-binding dependent) is required for promoter co-recruitment, or whether they are recruited independently. However, we believe that such detailed additional mechanistic studies are beyond the scope of the current manuscript (which already makes several robustly-supported new findings) and should be addressed separately in subsequent studies.

To summarise what we deem to be the main novel findings and significance of our study:

(i) the contribution of BRD9/ncBAF to IFN-stimulated gene expression and antiviral activity is a new finding that we thoroughly validate with multiple independent assays, several cell models, and different viral infection systems.

(ii) we provide evidence that BRD9 exhibits specificity with respect to the genes it impacts (e.g. only some ISGs, see Fig 4, and not TNF-α-induced genes, see new Fig EV2G and page 9, lines 184-186). Indeed, in our RNA-Seq data we noted very few gene expression changes upon loss of BRD9 (and loss of BRD9 does not impact general virus replication, which is heavily reliant on cellular genes) indicating that BRD9 is not a general regulator of all cellular transcription.

(iii) we show in new experiments using siRNAs to target ncBAF complex members (BRD9 and GLTSCR1) and PBAF complex members (BRD7 and ARID2) that the antiviral function of IFN is only impaired upon loss of ncBAF complex members, indicating that not all BAF complexes that mediate transcription play a role in innate immunity (new Fig EV1C). These data complement our existing data in Fig 5 using functional BRD7/9 domain swaps that we have now solidified with additional co-immunoprecipitation experiments (new Fig 5H). We believe that this concept of BAF complex specificity in innate immunity is novel.

(iv) we provide evidence for a new druggable component in the IFN pathway (BRD9), which expands the classes of target that could be inhibited in the future to treat some forms of autoinflammatory diseases. Current options are limited to targeting the JAK-family tyrosine kinases, therefore research to identify novel options is of great interest and significance to this field.

1st Revision - Editorial Decision 20th Jul 2021

Dear Prof. Hale,

Thank you for the submission of your revised manuscript to our editorial offices. I have now received the reports from the three referees that were asked to re-evaluate your study, you will find below. As you will see, the referees now support the publication of your study. However, all referees #2 and #3 have remaining points and suggestions to improve the manuscript I ask you to address in a final revised version of the manuscript. Please also provide a point-by-point-response addressing the remaining concerns of the referees.

Moreover, I have these editorial requests:

- Please make sure that the number "n" for how many independent experiments were performed, their nature (biological versus technical replicates), the bars and error bars (e.g. SEM, SD) and the test used to calculate p-values is indicated in the respective figure legends (also of the EV figures), and that statistical testing has been done where applicable. Please avoid the phrase 'independent experiment', but clearly state if these were biological or technical replicates. If statistical testing was done but there is no significant difference, please also mark this in the diagrams (n.s.).

- As the Western blots shown are significantly cropped, could you provide the source data for all the blots (main, EV figures). The source data will be published in a separate source data file online along with the accepted manuscript and will be linked to the relevant figure. Please submit the source data (scans of entire blots) together with the revised manuscript. Please include size markers for scans of entire blots, label the scans with figure and panel number and send one PDF file per figure.

- Please call out the single panels of Figs. EV4 and EV5 separately. Please check that all figure panels are called out separately and sequentially.

- Please remove the legends for the EV table and the datasets from the main manuscript file. For both file types, please put the legend on the first TAB of the respective excel files before uploading the final version.

- Finally, please find attached a word file of the manuscript text (provided by our publisher) with a few changes and queries we ask you to include in your final manuscript text. Please provide your final manuscript file with track changes, in order that we can see any modifications done.

In addition, I would need from you:
- a short, two-sentence summary of the manuscript (not more than 35 words).
- two to four bullet points highlighting the key findings of your study.
- a schematic summary figure (in jpeg or tiff format with the exact width of 550 pixels and a height of not more than 400 pixels) that can be used as a visual synopsis on our website.

I look forward to seeing the final revised version of your manuscript when it is ready. Please let me know if you have questions regarding the revision.

Kind regards,

Achim Breiling
Editor

EMBO Reports

Referee #1:

The authors have sufficiently addressed all my points. I have no further comments.

Referee #2:

I'm satisfied that the authors addressed most concerns and that the mechanism of BRD9 regulation of ISGs, although still not entirely clear, is further advanced with the additional experiments. A bromodomain mutant of BRD9 would really be beneficial to our mechanistic understanding so if the data could be readily provided, I would suggest including it in this manuscript. Otherwise, collectively it is an interesting body of work worthy of publication in EMBO reports.

Referee #3:

It was nice to see that the revised manuscript has clarified the specificity of the finding and significance of the study better. The manuscript has enhance the impact of the study greatly by focusing at the specificity of BRD9/ncBAF over PBAF. It was also nice to see the thorough discussion of the limits/caveats of the findings which in fact further clarified the novelty of the findings.

I agree that two new experiments provided in this revision, siRNA targeting for ncBAF vs PBAF (Fig EV1C) and the functional validation of the domain swapping mutants (fig 5H) help in solidifying the novelty and specificity of the study. Although the low impact of siRNA targeting on eliminating of anti-viral effect is bit concerning. Perhaps, including the siRNA effect on some of BRD9 target genes expression might complement this data.

I agree with the authors that the proposed ChIP-sequencing experiment might be beyond scope of the current study and the authors have demonstrated sufficient novelty and significance to be accepted for publication in EMBO reports.

Author response letter for EMBOR-2021-52823V2

We thank the referees for their constructive comments on our revised manuscript and for their positive assessment of its suitability for publication. Please find their comments below, with our responses indicated in blue italics.

Referee #1:

The authors have sufficiently addressed all my points. I have no further comments.

> We thank the referee for taking the time to reassess our manuscript.

Referee #2:

I'm satisfied that the authors addressed most concerns and that the mechanism of BRD9 regulation of ISGs, although still not entirely clear, is further advanced with the additional experiments. A bromodomain mutant of BRD9 would really be beneficial to our mechanistic understanding so if the data could be readily provided, I would suggest including it in this manuscript. Otherwise, collectively it is an interesting body of work worthy of publication in EMBO reports.

> We thank the referee for their positive assessment of our manuscript. Indeed, while we show in this manuscript that a bromodomain mutant of BRD9 that lacks the ability to bind acetyl-lysine residues (N216A) is inefficient at fully permitting IFN-stimulated antiviral activity (Figs. 5A & B), we do not yet know the identity of the acetylated target bound by BRD9. Identifying such a target will be an important aim of future studies.

Referee #3:

It was nice to see that the revised manuscript has clarified the specificity of the finding and significance of the study better. The manuscript has enhance the impact of the study greatly by focusing at the specificity of BRD9/ncBAF over PBAF. It was also nice to see the thorough discussion of the limits/caveats of the findings which in fact further clarified the novelty of the findings.

I agree that two new experiments provided in this revision, siRNA targeting for ncBAF vs PBAF (Fig EV1C) and the functional validation of the domain swapping mutants (fig 5H) help in solidifying the novelty and specificity of the study. Although the low impact of siRNA targeting on eliminating of anti-viral effect is bit concerning. Perhaps, including the siRNA effect on some of BRD9 target genes expression might complement this data.

I agree with the authors that the proposed ChIP-sequencing experiment might be beyond scope of the current study and the authors have demonstrated sufficient novelty and significance to be accepted for publication in EMBO reports.

> We thank the referee for their positive comments about our revised manuscript and its novelty and significance. It is possible that the low (though consistent) impact of the ncBAF-targeting siRNAs on reducing IFN-mediated antiviral activity is due to factors such as inefficient mRNA depletion or low turnover of the respective proteins leading to residual function. Such caveats of this experimental methodology are obviously overcome with our subsequent use/validation of the CRISPR/Cas9 knock-out system for BRD9 (3 independent clones) and the BRD9 degrader molecule, dBRD9-A. However, we fully acknowledge this caveat with our small siRNA screening experiment in Fig. EV1C, and have now added new text on page 8, lines 171-174 to highlight this limitation to the reader.

2nd Revision - Editorial Decision 27th Jul 2021

Prof. Benjamin Hale
University of Zurich
Institute of Medical Virology
Winterthurerstrasse 190
Zurich, Zurich 8057
Switzerland

Dear Prof. Hale,

I am very pleased to accept your manuscript for publication in the next available issue of EMBO reports. Thank you for your contribution to our journal.

At the end of this email I include important information about how to proceed. Please ensure that you take the time to read the information and complete and return the necessary forms to allow us to publish your manuscript as quickly as possible.

As part of the EMBO publication's Transparent Editorial Process, EMBO reports publishes online a Review Process File to accompany accepted manuscripts. As you are aware, this File will be published in conjunction with your paper and will include the referee reports, your point-by-point response and all pertinent correspondence relating to the manuscript.

If you do NOT want this File to be published, please inform the editorial office within 2 days, if you have not done so already, otherwise the File will be published by default [contact: emboreports@embo.org]. If you do opt out, the Review Process File link will point to the following statement: "No Review Process File is available with this article, as the authors have chosen not to make the review process public in this case."

Should you be planning a Press Release on your article, please get in contact with emboreports@wiley.com as early as possible, in order to coordinate publication and release dates.

Thank you again for your contribution to EMBO reports and congratulations on a successful publication. Please consider us again in the future for your most exciting work.

Yours sincerely,

Achim Breiling
Editor
EMBO Reports

****

THINGS TO DO NOW:

You will receive proofs by e-mail approximately 2-3 weeks after all relevant files have been sent to

our Production Office; you should return your corrections within 2 days of receiving the proofs.

Please inform us if there is likely to be any difficulty in reaching you at the above address at that time. Failure to meet our deadlines may result in a delay of publication, or publication without your corrections.

All further communications concerning your paper should quote reference number EMBOR-2021-52823V3 and be addressed to emboreports@wiley.com.

Should you be planning a Press Release on your article, please get in contact with emboreports@wiley.com as early as possible, in order to coordinate publication and release dates.

EMBO PRESS

YOU MUST COMPLETE ALL CELLS WITH A PINK BACKGROUND ↓

PLEASE NOTE THAT THIS CHECKLIST WILL BE PUBLISHED ALONGSIDE YOUR PAPER

Corresponding Author Name: Benjamin G. Hale
Journal Submitted to: EMBO Reports
Manuscript Number: EMBOR-2021-52823-T

USEFUL LINKS FOR COMPLETING THIS FORM

http://www.antibodypedia.com
http://1degreebio.org
http://www.equator-network.org/reporting-guidelines/improving-bioscience-research-repor
http://grants.nih.gov/grants/olaw/olaw.htm
http://www.mrc.ac.uk/Ourresearch/Ethicsresearchguidance/Useofanimals/index.htm
http://ClinicalTrials.gov
http://www.consort-statement.org
http://www.consort-statement.org/checklists/view/32-consort/66-title
http://www.equator-network.org/reporting-guidelines/reporting-recommendations-for-tumo
http://datadryad.org
http://figshare.com
http://www.ncbi.nlm.nih.gov/gap
http://www.ebi.ac.uk/ega
http://biomodels.net/
http://biomodels.net/miriam/
http://jjj.biochem.sun.ac.za
http://oba.od.nih.gov/biosecurity/biosecurity_documents.html
http://www.selectagents.gov/

Reporting Checklist For Life Sciences Articles (Rev. June 2017)

This checklist is used to ensure good reporting standards and to improve the reproducibility of published results. These guidelines are consistent with the Principles and Guidelines for Reporting Preclinical Research issued by the NIH in 2014. Please follow the journal's authorship guidelines in preparing your manuscript.

A- Figures
1. Data
The data shown in figures should satisfy the following conditions:

- the data were obtained and processed according to the field's best practice and are presented to reflect the results of the experiments in an accurate and unbiased manner.
- figure panels include only data points, measurements or observations that can be compared to each other in a scientifically meaningful way.
- graphs include clearly labeled error bars for independent experiments and sample sizes. Unless justified, error bars should not be shown for technical replicates.
- if n< 5, the individual data points from each experiment should be plotted and any statistical test employed should be justified
- Source Data should be included to report the data underlying graphs. Please follow the guidelines set out in the author ship guidelines on Data Presentation.

2. Captions

Each figure caption should contain the following information, for each panel where they are relevant:

- a specification of the experimental system investigated (eg cell line, species name).
- the assay(s) and method(s) used to carry out the reported observations and measurements
- an explicit mention of the biological and chemical entity(ies) that are being measured.
- an explicit mention of the biological and chemical entity(ies) that are altered/varied/perturbed in a controlled manner.

- the exact sample size (n) for each experimental group/condition, given as a number, not a range;
- a description of the sample collection allowing the reader to understand whether the samples represent technical or biological replicates (including how many animals, litters, cultures, etc.).
- a statement of how many times the experiment shown was independently replicated in the laboratory.
- definitions of statistical methods and measures:
  - common tests, such as t-test (please specify whether paired vs. unpaired), simple χ2 tests, Wilcoxon and Mann-Whitney tests, can be unambiguously identified by name only, but more complex techniques should be described in the methods section;
  - are tests one-sided or two-sided?
  - are there adjustments for multiple comparisons?
  - exact statistical test results, e.g., P values = x but not P values < x;
  - definition of 'center values' as median or average;
  - definition of error bars as s.d. or s.e.m.

Any descriptions too long for the figure legend should be included in the methods section and/or with the source data.

In the pink boxes below, please ensure that the answers to the following questions are reported in the manuscript itself. Every question should be answered. If the question is not relevant to your research, please write NA (non applicable). We encourage you to include a specific subsection in the methods section for statistics, reagents, animal models and human subjects.

B- Statistics and general methods

Please fill out these boxes ↓ (Do not worry if you cannot see all your text once you press return)

1.a. How was the sample size chosen to ensure adequate power to detect a pre-specified effect size?	No sample-size calculations were performed. Sample size was determined to be adequate based on the magnitude and consistency of measurable differences between groups
1.b. For animal studies, include a statement about sample size estimate even if no statistical methods were used.	NA
2. Describe inclusion/exclusion criteria if samples or animals were excluded from the analysis. Were the criteria pre-established?	NA
3. Were any steps taken to minimize the effects of subjective bias when allocating animals/samples to treatment (e.g. randomization procedure)? If yes, please describe.	No
For animal studies, include a statement about randomization even if no randomization was used.	NA
4.a. Were any steps taken to minimize the effects of subjective bias during group allocation or/and when assessing results (e.g. blinding of the investigator)? If yes please describe.	No
4.b. For animal studies, include a statement about blinding even if no blinding was done	NA
5. For every figure, are statistical tests justified as appropriate?	Yes. Statistical analyses were performed in GraphPad Prism 9.0.0.
Do the data meet the assumptions of the tests (e.g., normal distribution)? Describe any methods used to assess it.	Statistical analyses were performed in GraphPad Prism 9.0.0. We did not independently assess test assumptions
Is there an estimate of variation within each group of data?	Yes

Is the variance similar between the groups that are being statistically compared?	Yes

C- Reagents

6. To show that antibodies were profiled for use in the system under study (assay and species), provide a citation, catalog number and/or clone number, supplementary information or reference to an antibody validation profile. e.g., Antibodypedia (see link list at top right), 1DegreeBio (see link list at top right).	The catalog number and supplier for each commercial antibody used is given in the Materials and Methods section of the manuscript. No additional validation was performed beyond the supplier's data, which is available from the appropriate website
7. Identify the source of cell lines and report if they were recently authenticated (e.g., by STR profiling) and tested for mycoplasma contamination.	The source of each cell-line is indicated in the manuscript, along with the following statement: 'Cell-lines were not authenticated, but were routinely tested for mycoplasma contamination (GATC, Germany). None of the cell-lines used ever tested positive for mycoplasma'

* for all hyperlinks, please see the table at the top right of the document

D- Animal Models

8. Report species, strain, gender, age of animals and genetic modification status where applicable. Please detail housing and husbandry conditions and the source of animals.	NA
9. For experiments involving live vertebrates, include a statement of compliance with ethical regulations and identify the committee(s) approving the experiments.	NA
10. We recommend consulting the ARRIVE guidelines (see link list at top right) (PLoS Biol. 8(6), e1000412, 2010) to ensure that other relevant aspects of animal studies are adequately reported. See author guidelines, under 'Reporting Guidelines'. See also: NIH (see link list at top right) and MRC (see link list at top right) recommendations. Please confirm compliance.	NA

E- Human Subjects

11. Identify the committee(s) approving the study protocol.	NA
12. Include a statement confirming that informed consent was obtained from all subjects and that the experiments conformed to the principles set out in the WMA Declaration of Helsinki and the Department of Health and Human Services Belmont Report.	NA
13. For publication of patient photos, include a statement confirming that consent to publish was obtained.	NA
14. Report any restrictions on the availability (and/or on the use) of human data or samples.	NA
15. Report the clinical trial registration number (at ClinicalTrials.gov or equivalent), where applicable.	NA
16. For phase II and III randomized controlled trials, please refer to the CONSORT flow diagram (see link list at top right) and submit the CONSORT checklist (see link list at top right) with your submission. See author guidelines, under 'Reporting Guidelines'. Please confirm you have submitted this list.	NA
17. For tumor marker prognostic studies, we recommend that you follow the REMARK reporting guidelines (see link list at top right). See author guidelines, under 'Reporting Guidelines'. Please confirm you have followed these guidelines.	NA

F- Data Accessibility

18: Provide a "Data Availability" section at the end of the Materials & Methods, listing the accession codes for data generated in this study and deposited in a public database (e.g. RNA-Seq data: Gene Expression Omnibus GSE39462, Proteomics data: PRIDE PXD000208 etc.) Please refer to our author guidelines for 'Data Deposition'.<br><br>Data deposition in a public repository is mandatory for:<br>a. Protein, DNA and RNA sequences<br>b. Macromolecular structures<br>c. Crystallographic data for small molecules<br>d. Functional genomics data<br>e. Proteomics and molecular interactions	This information is available at the end of the Materials and Methods section of the manuscript, where accession codes have been listed
19. Deposition is strongly recommended for any datasets that are central and integral to the study; please consider the journal's data policy. If no structured public repository exists for a given data type, we encourage the provision of datasets in the manuscript as a Supplementary Document (see author guidelines under 'Expanded View' or in unstructured repositories such as Dryad (see link list at top right) or Figshare (see link list at top right).	The datasets are deposited in the appropriate public repositories and the accession codes are listed in the manuscript
20. Access to human clinical and genomic datasets should be provided with as few restrictions as possible while respecting ethical obligations to the patients and relevant medical and legal issues. If practically possible and compatible with the individual consent agreement used in the study, such data should be deposited in one of the major public access-controlled repositories such as dbGAP (see link list at top right) or EGA (see link list at top right).	NA
21. Computational models that are central and integral to a study should be shared without restrictions and provided in a machine-readable form. The relevant accession numbers or links should be provided. When possible, standardized format (SBML, CellML) should be used instead of scripts (e.g. MATLAB). Authors are strongly encouraged to follow the MIRIAM guidelines (see link list at top right) and deposit their model in a public database such as Biomodels (see link list at top right) or JWS Online (see link list at top right). If computer source code is provided with the paper, it should be deposited in a public repository or included in supplementary information.	NA

G- Dual use research of concern

22. Could your study fall under dual use research restrictions? Please check biosecurity documents (see link list at top right) and list of select agents and toxins (APHIS/CDC) (see link list at top right). According to our biosecurity guidelines, provide a statement only if it could.	No
